# Availability of drugs for the treatment of multidrug-resistant/rifampicin-resistant tuberculosis in the World Health Organization European Region, October 2023

**DOI:** 10.2807/1560-7917.ES.2024.29.17.2400211

**Published:** 2024-04-25

**Authors:** Ralf Otto-Knapp, Suzanne Edwards, Giorgi Kuchukhidze, Stefan Kröger, Brit Häcker, Stela Bivol, Askar Yedilbayev

**Affiliations:** 1 German Central Committee against Tuberculosis (DZK), Berlin, Germany; 2 Independent Consultant & Department of Healthcare Management, Technical University of Berlin, Berlin, Germany; 3 World Health Organization, Regional Office for Europe, Copenhagen, Denmark; 4 Infectious Disease Epidemiology, Robert Koch Institute, Berlin, Germany

**Keywords:** tuberculosis, MDR/RR-TB, drug availability, access, Europe, pretomanid

## Abstract

The BPaLM regimen (bedaquiline, pretomanid, linezolid and moxifloxacin) recently recommended by the World Health Organization offers short, safe, and effective treatment for multidrug-resistant/rifampicin-resistant tuberculosis (TB). In a survey with national TB focal points in 18 central and western European countries to explore barriers for the implementation of BPaLM, only three reported full availability of pretomanid, a necessary component of this regimen. Implementation barriers included financing and procurement. Solutions on national and supranational level are needed to guarantee universal access.

Comprising 53 countries, the World Health Organization (WHO) European Region includes countries with the highest multidrug-resistant/rifampicin-resistant (MDR/RR) tuberculosis (TB) rates observed globally, and countries with some of the world’s lowest TB rates [1]. In 2022, population displacement in Europe triggered by the war against Ukraine has led to an increase in MDR/RR-TB notifications in many countries in western and central Europe and brought into focus challenges in treatment continuity and regional inequities in access to TB medicines [2,3]. The uptake of effective regimens recommended by the latest WHO MDR/RR TB guidelines [4] is affected by drug availability challenges. With a growing number of MDR/RR-TB patients in Europe, the WHO Regional Office for Europe conducted a survey to explore common and country-specific challenges and calls for efforts to improve the situation.

## The new drug-resistant tuberculosis treatment

In 2020, the WHO began recommending fully oral treatment regimens for the treatment of drug-resistant (DR) TB. These render therapy more safe, effective and person-centred, with shorter treatment durations [4,5]. In its latest guidelines, the WHO recommends a short 6-month BPaLM regimen comprised of bedaquiline (Bdq), pretomanid (Pa), linezolid (L) and moxifloxacin (Mfx) as the preferred treatment option for patients with MDR/RR-TB, over the use of 9-month and longer regimens [4]. This change has the potential to improve treatment success rates for MDR/RR-TB, particularly in the WHO European Region, where treatment is only successful in 55% of cases, below all other WHO Regions [6]. The new regimen could offer the additional benefit of reduced health system costs and decreased stigma associated with lengthy DR-TB treatment. Moreover, many individuals affected by DR-TB belong to highly mobile and vulnerable populations, for whom shorter regimens offer substantial benefits.

Despite these benefits reports have been emerging that countries with relatively low TB burden are struggling to secure the necessary components of the WHO-recommended DR-TB regimens [7]. To advance efforts towards TB elimination, shorter regimens and tolerable drugs in convenient formulations are needed, especially in case of MDR/RR-TB. The public health risk posed by untreated, improperly, or interruptedly treated individuals with DR-TB is raising concerns among physicians, public health experts and policymakers [4,8]. Notably, attracting mention in the political declaration from the United Nations high-level meeting on TB September 2023 which specifically called for ‘’universal availability” of the medicines central to these regimens [9].

## Tuberculosis treatment supplies

In the WHO European Region, two main supply channels meet national health systems’ demand for TB treatments. In western and central Europe, a plethora of small generic companies supply the older single-component medicines. Newer, patent-protected medicines are centrally registered and available through international producers. With a low number of patients fragmented across diverse health systems, this market is economically unattractive for suppliers and in many cases, the market will only support a single supplier [10].

In eastern Europe where the TB burden is higher and countries are eligible for donor support, the Stop TB Partnership’ Global Drug Facility (GDF) provides a donor-subsidised one-stop shop for all TB treatment needs, offering favourable prices [11]. In western Europe, GDF’s concessionary prices are not accessible as international manufacturers typically register and offer newer, patent-protected DR-TB medicines for a standard price across EU/EEA countries, which is substantially higher than that offered to countries by GDF. This standard pricing model does not always consider a country’s purchasing power, causing pricing barriers for many European countries that experience an increase in MDR/RR-TB notifications as a consequence of the humanitarian crisis.

## Survey on countries’ ability to implement the BPaLM regimen 

A systematic overview at the programmatic level on availability and barriers to access MDR/RR-TB treatments in European countries non supported by GDF is lacking; therefore, in October 2023, the WHO Regional Office for Europe undertook a survey to better understand countries’ ability to implement the new treatment approaches and gain insights into the current challenges related to accessing recommended DR-TB treatment components.

This survey was distributed to 29 countries in central and western Europe, with the objective of gathering both quantitative and qualitative data on availability and pricing of medicines used in MDR/RR-TB treatment. Specifically, the survey explored the barriers to implementing the new BPaLM regimen, with a focus on aspects of drug procurement, financing within national health systems, drug supply and anticipated future demand. The questionnaire was developed using Microsoft Forms, and the collected information was subsequently analysed using descriptive statistics. Ethical approval was not required for this study; however, publication consent was obtained during the survey.

## Current MDR/RR-TB drug availability

**Figure 1:**
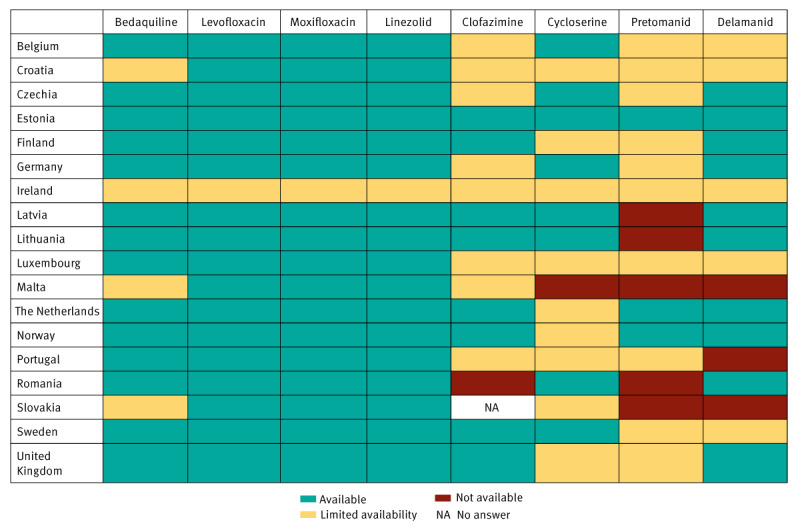
Categorical answers to the question ‘Indicate availability of the following medicines for treatment of MDR/RR-TB in your setting/country’, WHO Regional Office for Europe survey, central and western Europe, October 2023 (n = 18 countries)

MDR: multidrug-resistant; RR: rifampicin-resistant; TB: tuberculosis; WHO: World Health Organization.

Eighteen of the 29 countries provided responses. The respondents were WHO-TB focal points appointed by their respective national health authorities. Regarding unconditional availability of DR-TB drugs, all respondent countries indicated no issues with levofloxacin, moxifloxacin and linezolid, except Ireland. For other key regimen components, availabilities in the respondent countries were reported as follows: bedaquiline in 14 of 18 countries, delamanid (Dlm) in 10 of 18 countries, clofazimine (Cfz) in eight of 17 countries, cycloserine (Cs) in eight of 18 countries, and pretomanid in three of 18 countries (Figure 1).

Various reasons were reported for the limited availability of certain medicines (open question, qualitative data), including transient or current stock-outs (Cfz, Cs) and slow and bureaucratic importation procedures (Cfz, Cs, Dlm, Pa). Delays in procurement attributed to mandatory approval processes by hospital medicines committees (Bdq, Cfz, Cs, Dlm, Pa) or ethical committees (Pa) were identified as access barriers. In countries where no availability was reported, a lack of marketing authorisation was cited as the primary barrier to access (Cfz, Dlm, Pa), although this could reflect challenges with these products’ labels and licensing arrangements.

**Figure 2:**
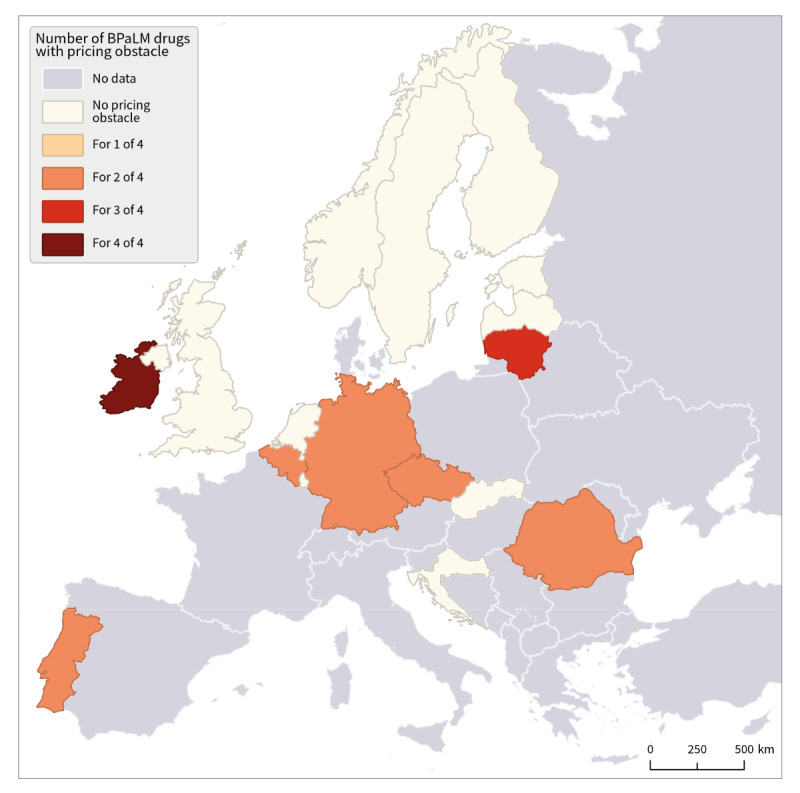
Categorical answers to the question ‘Is the price of the following drugs in your country an obstacle for the implementation of the new WHO recommendation on BPaL(M)?’ WHO Regional Office for Europe survey, central and western Europe, October 2023 (n = 18 countries)

Pricing was identified as a frequently named obstacle for the implementation of the new WHO recommended BPaLM regimen for bedaquiline in seven of 18 countries, for pretomanid in five of 17 countries, and less frequently for linezolid in three of 18 and moxifloxacin in two of 18 countries (Figure 2). Other commonly reported challenges for BPaLM implementation (open question, qualitative data) included drug availability, procurement procedures and relatively small numbers of DR-TB cases in the responding countries.

BPaLM: regimen comprised of bedaquiline (Bdq), pretomanid (Pa), linezolid (L) and moxifloxacin (Mfx).

Data source: WHO Regional Office for Europe. Produced by: WHO Regional Office for Europe.

The demand for DR-TB drugs included in BPaLM was projected to increase in eight of nine countries that provided specific responses to this open question. Many countries reported a rise in the number of MDR/RR-TB notifications, with migration, particularly from Ukraine, cited as a contributing factor in some countries. Nonetheless, in most participating countries, the number of MDR/RR-TB cases were reported to be relatively small, with 12 of 18 countries reporting fewer than 15 cases of MDR/RR-TB in 2022.

## Discussion

The relatively low volumes of TB medicines required by low-incidence countries in western Europe compared with high-burden countries in Eastern Europe and Central Asia, present their own challenges for ensuring and sustaining access and availability, i.e. pricing and low-purchase volumes. However, our survey results indicate challenges across health system functions. Unless conditions changed after the survey, our results indicate the need for European countries to reevaluate their current health system procedures, specifically procurement, pricing and reimbursement, to meet the rising demand and ensure access to medicines, particularly those comprising the newly recommended MDR/RR-TB regimens and remove identified barriers to leading to their limited availability. This could be mitigated by exploring alternative procurement mechanisms at the country or pan-European level to address specific product gaps and by finding strategies to ensure access to modern TB medicines, thereby contributing to a sustainable response to current and future emergencies.

Our findings on the availability of MDR-TB medicines on a programmatic level are supported by a recent survey of the Tuberculosis Network European Trials group (TBNET) [12]. It is of concern that 54.5% of respondents representing clinicians in metropolitan areas of a considerable portion of WHO European Region countries (44/54) reported a lack of access to pretomanid. The implications are serious: if any component of the regimen is unavailable, patients may be forced to switch to longer, less tolerable treatment regimens lasting 18 months or longer. This not only affects individual patient outcomes but also brings extra burden to national health systems and hampers efforts to eliminate TB effectively [4].

Importantly, the number of MDR/RR-TB cases is low in most countries covered by our survey, which may limit political commitment to ensure drug availability for this rare disease [6]. Markets for MDR-TB drugs are small in these European countries limiting the interest for domestic licensing or production. This also concerns other important TB drugs like rifapentine as well as child friendly drug formulations and combination tablets [13,14]. Decreasing case numbers in the context of TB elimination in Europe could inadvertently worsen conditions at European markets.

## Conclusion

The WHO-recommended short regimen was a long-awaited breakthrough in the treatment and care of MDR/RR-TB globally and has improved treatment outcomes considerably. According to our survey results, currently only three of 18 central and western European countries reported unconditional availability to pretomanid, a key component to the preferred BPaLM regimen. High drug costs for bedaquiline and pretomanid were identified as barriers for the implementation of BPaLM. Other barriers related to procurement and financing of the new drugs varied from country to country, reflecting the specific conditions within each country's healthcare system. Under these conditions, the implementation of the WHO-recommended short regimen for MDR/RR-TB treatment is not guaranteed across the WHO European Region. National and supranational solutions are needed to ensure the availability of this regimen for all eligible patients within every healthcare system in Europe to re-catalyse efforts towards TB elimination goals. Exploring potential synergies to address the reported barriers to full availability of MDR/RR-TB drugs in the European Region could be beneficial.

## Disclaimer

All references to Kosovo in this document should be understood to be in the context of the United Nations Security Council resolution 1244 (1999). The designations employed and the presentation of the material in this publication do not imply the expression of any opinion whatsoever on the part of WHO concerning the legal status of any country, territory, city or area or of its authorities, or concerning the delimitation of its frontiers or boundaries. Dotted and dashed lines on maps represent approximate border lines for which there may not yet be full agreement.
